# Threads Made with Blended Biopolymers: Mechanical, Physical and Biological Features

**DOI:** 10.3390/polym11050901

**Published:** 2019-05-17

**Authors:** Annamaria Visco, Cristina Scolaro, Alberto Giamporcaro, Salvatore De Caro, Elisabetta Tranquillo, Michelina Catauro

**Affiliations:** 1 Department of Engineering, University of Messina, C.da Di Dio, 98166 Messina, Italy; cscolaro@unime.it (C.S.); alberto.giamporcaro@gmail.com (A.G.); sdecaro@unime.it (S.D.C.); 2 Institute for Chemical-Physical Processes CNR –IPCF, Viale Ferdinando Stagno d’Alcontres, 37, 98158 Messina, Italy; 3 Department of Engineering, University of Campania Luigi Vanvitelli, VialeAbramo Lincoln 5, 81100 Caserta, Italy; elisabetta.tranquillo@unicampania.it (E.T.); michelina.catauro@unicampania.it (M.C.)

**Keywords:** biopolymer blend, thread, mechanical characterization, bioactivity, antibacterial

## Abstract

Poly (Lactic Acid), PLA, and Poly (ε-CaproLactone), PCL, compatibilized with Ethyl Ester l-Lysine Triisocyanate (LTI) can be employed as biomaterials. We mixed PLA with PCL and LTI in a twin extruder and by a melt spinning process obtained threads with an average diameter of about 0.3 mm. In order to study the possible application of these threads, mechanical tensile (with the calorimetric and morphological investigations) and biological tests were performed. The results highlighted these biopolymers as promising materials for sutures since they can be rigid and elastic (especially by increasing the PCL amount in the blend), and they are bioactive, able to inhibit bacterial growth. This paper represents a starting point to optimize the blend composition for biomedical suture application.

## 1. Introduction

Thermoplastic biopolymers such as Poly(Lactic Acid), PLA, and Poly (ε-CaproLactone), PCL, can be employed as biomaterials in regenerative medicine and drug delivery systems (i.e., bone and nerve regeneration, scaffolds, sutures, etc.) [1]. They are bio-compatible, biodegradable, bioactive and have complementary physical-mechanical-biological features: PLA is sensitive to thermal degradation, to enzymatic and chemical hydrolysis and has a good dissolution time [2], while PCL has higher thermal stability and longer degradation times than PLA.

PLA exhibited a high yield strength and stiffness, but it pays in ductility, being quite brittle at room temperature. The glass transition temperature (*T_g_*) of PLA is of +60 °C and it thermally degrades at about 235 °C. PCL exhibits high ductility; it is a rubber-like polymer (*T_g_* = −60 °C) with good stability under ambient conditions and under thermo-mechanical stress, and its workability is much greater than that of PLA since it only starts to degrade at *T* > 360 °C [3].

It is worth mentioning the reason of the choice of PCL as a complementary material for PLA, considered as one of the most significant bio-degradable biopolymers which have great potential to replace the traditional petroleum-based polymers [4]. To overcome the low toughness and brittleness of PLA it could be blended with other biodegradable polymers from microbial fermentation such as (PolyHydroxyButyrate, PHB). However, on the one hand, PHB improves the impact strength of pure PLA, but on the other hand, PHB has a low thermal stability [5]. Similarly, PLA could be blended with CS (ChitoSan) or with PEG (PolyEthylene Glycol); they are both hydrophilic materials with fast degradation times, but with a poor mechanical strength. Instead, PCL is a rubbery polymer with good ductility because of its low glass transition temperature and also a good thermal stability. This makes PCL suitable for improving the features of PLA, especially from a mechanical point of view.

The complementary physical and mechanical features of PCL and PLA require the combination of the two materials [6]. Because of their different chemical structure, they are not miscible [7] and need a suitable compatibilizing agent. Possible ways to achieve a good blending effect is the addition of a pre-made polymeric compatibilizer, reactive blending and exposure to high energy radiation [8]. Premade compatibilizing agents are di-block or tri-block copolymers [4,9]. However, they represent an additional cost for the copolymer synthesis and additional process time necessary to reach the right balance of the blocks for a given blend [7]. On the other hand, the exposure to radiation needs a specific and appropriate high energy source, such as an accelerated electron beam [8]. A new concept of compatibilization by using inorganic nanoparticles (such as titanium dioxide, silica, zinc oxide, etc.) that stabilizes the two immiscible blends at their interfaces has also been proposed [5]. In this paper, we considered the chemical compatibilization by the addition of Ethyl Ester l-Lysine Triisocyanate (LTI); it induces a reactive blending process resulting in a branched and/or cross-linked co-polyester-urethane network [10,11,12]. In a previous paper, we have already studied PLA and PCL compatibilized with LTI and blended in a Brabender mixing; LTI effectively enhances the dispersion of the two phases with a synergistic effect on the mechanical features that can be tailored (in terms of stiffness and ductility) by changing the blend composition [3]. In this paper, we studied the physical and mechanical features of the PLA/PCL blends 50/50 *w*/*w*), with different amounts of LTI as compatibilizing agent (from 0.5 phr to 5 phr, where phr stands for: parts per hundred resin/rubber), processed in a twin extruder and then stretched by a melt spinning process in order to obtain threads. The resulting threads have been characterized by several tests (physical, mechanical and biological) in order to verify the possible applications of these bio-polyester blends as sutures.

A suture is a filament used in surgery to merge biological tissues, distinguished as absorbable (usually used for the suturing of internal structures) or non-absorbable (usually used for skin suturing), which require removal because they are not degraded by the body [13]. The absorbing mechanism is a polymeric degradation that occurs by slow hydrolysis in tissue fluids with minimal tissue reaction [14].

Besides, sutures can be natural (derived from the animal kingdom, such as catgut, silk, collagen, or vegetable, flax/cotton) or artificial, which are derived from organic materials and synthetic threads, such as polypropylene, polyamides, polyesters [15].

Relatively recent literature studies focused their attention on materials useful for sutures with improved features such as the capability to release drugs to accelerate wound healing and prevent wound infections [13,16,17,18]. The ideal suture must be not only biocompatible, avoiding possible inflammatory responses when implanted into the body, but it should have the appropriate time of bio-degradability, good mechanical features (stiffness, deformability and form a secure knot) [19]. Commercial absorbable sutures are generally based on guts, polyglactin, polydioxane, polyglytone, polyglyconate, polyglecaprone and glycomer, while non-absorbable ones are based on silk, polypropylene, nylon and polybutester [20]. Visco-elastic plastic features of sutures made of PLA/PCL (90/10) have been studied by Vieira et al. [21] while He at al. studied sutures of PLLA [16]. Sutures based on polyhydroxyalkanoates (PHA) have been studied by Shishatskaya et al. [22], who observed no inflammatory response by the tissues of laboratory animals into which they have implanted.

To date, there is no scientific study in the literature on the application of PLA/PCL/LTI blends as sutures. A good biological interaction of co-polyester-urethane networks from biodegradable polyesters and Lysine Isocyanate compounds has been proven by some other studies [23,24]. For these reasons, we started this characterization of our threads by tensile mechanical features correlated with a calorimetric and a morphological investigation, and finally, checking their bioactivity and antibacterial activity. This is a preliminary investigation to verify the possible application of PLA/PCL/LTI blends as sutures; encouraging results will be improved at a later time by optimizing the material composition specifically for this application. Due to the opposite features of PLA and PCL mentioned earlier, in this paper, we considered the blend made by the same amount of them: PLA/PCL 50/50 ratio (*w*/*w*) in which all the physical and mechanical properties were exactly balanced. In addition, we know from our previous study that if we need to favor the material’s deformability and toughness and prolong the degradation time, we must consider a blend rich in PCL (such as the PLA/PCL 20/80 ratio *w*/*w*) already studied by Visco et al. [3]. Similarly, if we need to enhance the stiffness (shortening the degradation time), we must consider a blend rich in PLA (such as the PLA/PCL 80/20 ratio *w*/*w*) already studied by Visco et al. [3]).

## 2. Materials and Methods

### 2.1. Materials

Blends were obtained by adding the reactive compatibilizing agent in various amounts to a 50/50 ratio (*w*/*w*) of PLA/PCL (where PLA stands for the Poly(Lactic Acid) BioFlex 6510, supplied by FKUR (Kunststoff, GmbH Willich, Germany), *M*_n_ = 197,000 Da and PCL stands for Poly (ε-CaproLactone), supplied by Sigma-Aldrich s.r.l. (Milan, Italy) *M*_n_ = 80,000 Da, both dried at 50 °C overnight before processing).

The two immiscible polymers have been compatibilized by adding the Ethyl Ester l-Lysine Triisocyanate (LTI, purchased from Infine Chemicals Ltd., Pudong Avenue, Shangai, China, *d* = 1.21 g/mL) in different amounts (0.5–1.0–2.0–5.0 phr, per hundred resin). The threads have been codified as EX (for the EXstruded uncompatibilized PLA/PCL thread,) and as EXT05, EXT1, EXT2 and EXT5 for the Extruded compatibilized PLA/PCL/LTI threads, depending on the LTI amounts (code “T”) of 0.5–1.0–2.0–5.0 phr, respectively.

PLA/PCL and PLA/PCL/LTI blends have been extruded in a Mini Lab II Haake Rheomex CTW 5 conical twin-screw extruder at 160 °C with a screw speed of 20 rpm for a re-circulating time of 3 min and extrusion time of 8 min. The temperature of 160 °C was chosen since a lower temperature (150 °C) was not enough to completely melt the PLA while a higher temperature (180 °C) degrades the polymers inside the extruder. LTI was added 2 min after the mixing started.

**Figure 1:**
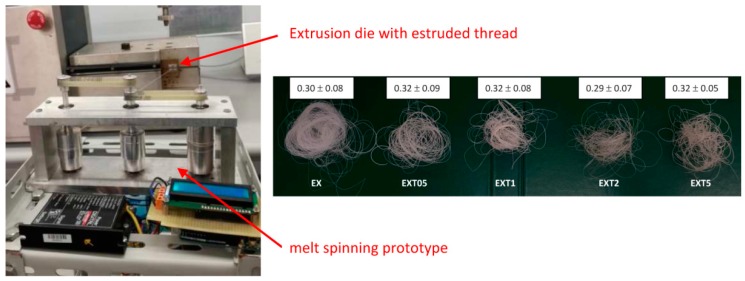
Thespinning process for thread production (**left**); the image of the threads, with the average diameter expressed in mm (**right**).

The monofilament that comes out of the extrusion die is quenched in air and has a diameter of 1.5 mm. Then, it is stretched at a constant rate of 67 rpm by means of three pulleys of the spinning prototype system that is able to collect the wire (Figure 1, left). Thus, the filament thread reaches an average diameter of about 0.3 mm (Figure 1, right). For the transmission of the motion of the mechanical shafts, toothed belts have been chosen and suitably assembled with the pulleys. The prototype is equipped with a high quality brushless DC motor (BLDC). The speed of the BLDC motor is controlled using a Digital EC Controller (DEC). The value of 0.3 mm is within the typical diameter range of the suture threads described in the European Pharmacopeia (metric scale). The European Pharmacopeia identifies the wires regardless of their nature and characteristics, with a single numbering corresponding to their caliber expressed in tenths of a millimeter which ranges from 0.1 (minimum caliber) to 8, (maximum caliber) or from 0.02 mm to 0.89 mm [25]. In Figure 1 (right), we can observe a change in color of the blend from the typical white-milk (of PLA/PCL) to light-brown (of PLA/PCL/LTI) by increasing the LTI amount, whose color is dark brown.

### 2.2. Characterization and Testing

Rheological measurements allow us to check the viscosity (η) change during the PLA, PCL and LTI reactive mixing by means of MiniLab II Haake Rheomex CTW 5, at a temperature of 160 °C, with a screw rate ranging between 0.1 and 100 rpm (shear rate γ’) for a re-circulating time of about 15 min.

Differential Scanning Calorimetry was performed by a Mettler Toledo-DSC1, from room temperature to 200 °C, with a heating rate of 10 °C/min and water cooling.

The crystalline degree of PCL, *χ*, was calculated using the following equation:

(1) $$\chi =\;\frac{\Delta H_{m}}{\varphi \cdot \Delta H_{0}}$$

where Δ*H_m_* [J/g] is the melting enthalpy, *φ* is the weight fraction of the studied material and Δ*H*_0_ [J/g] is the theoretical enthalpy of fusion of a polymer crystal of infinite extension [26,27]. The initial crystalline fraction of PLA, *χ_i_*, was calculated using the following equation:

(2) $$\chi_{i}=\;\frac{\Delta H_{m}+\Delta H_{cc}}{\varphi \cdot \Delta H_{0}}$$

where Δ*H_cc_* [J/g] is the “cold-crystallization” enthalpy of PLA. Cold crystallization is due to the PLA re-organization into more ordered regions induced by the thermal heating of the DSC test [28]. Equation (3) has been used to calculate the crystalline degree of the cold crystallized PLA (*χ*_cc_).

(3) $$\chi_{cc}=\;\frac{\Delta H_{cc}}{\varphi \cdot \Delta H_{0}}$$

Mechanical uniaxial tensile tests were conducted by a Lloyd LR10K universal testing machine, following the standard ASTM D2256-2002 with a length of about 500 mm as the distance of the clamps along the specimen axis (active length), a crosshead speed of testing of 10 mm/min, a load cell of 500 N, a room temperature of about 20 °C, and a relative humidity of about 51% (it must be no more than 65%). The threads were tested in the straight mode by measuring the average tensile parameter and their standard deviations of yielding strength (σ*_y_*, MPa), breaking strength (σ*_b_*, MPa), breaking strain (*_b_*, %), Young modulus (*E*, MPa), work at break (J).

Mechanical comparison with the data of the threads from the literature were performed on the EXT1 sample (chosen as the best among those investigated) using 600 mm as the active length and a crosshead speed of testing of 120 mm/min according to the United States Pharmacopeia (USP) standards for tensile strength (both in straight, unknotted and in o-knot, knotted) [20]. The o-knot was placed at the center of the active length. Threads were attached to each tensile mount by tying the suture to the crossbar of the tensile mount and then wrapping two times along the shaft of the tensile mount.

Scanning Electron Microscopy (SEM) images were obtained using an FEI Quanta FEG 450 microscope operating with an accelerating voltage of 5 kV in the low vacuum mode and placing the samples on aluminum holders by means of graphitic glue. Threads were broken in liquid nitrogen to obtain the inner circular section observed by the microscope. Samples surfaces were coated with a thin chromium layer to make them conductive. Magnifications were of 7250× and 20k×.

A Bioactivity Testproposed by Kokubo [29] was used to evaluate the bioactive properties of the materials. The samples were soaked in a simulated body fluid (SBF) for 7, 14, 21 days at 37 °C. The ion concentration contained in the SBF solution is almost equal to that of human blood plasma (Table 1). The SBF was prepared by dissolving different chemical reagents; NaCl, NaHCO_3_, KCl, MgCl_2_, 1 M HCl, CaCl_2_·6H_2_O, and Na_2_SO_4_ (Sigma-Aldrich, St. Louis, MO, USA) as shown in Table 1. 1 M HCl was used to adjust the pH of the buffer to pH 7.4.

The solution was replaced every two days to avoid the depletion of ionic species in the SBF. After 7, 14 and 21 days, the samples were dried in a glass desiccator and, afterward, the formation of an apatite layer on the sample surface was evaluated by SEM and XRD analysis. XRD analysis was carried out in the range of 2*θ* from 20° to 70° using a Philips 139 diffractometer (Philips, Amsterdam, the Netherlands) equipped with a PW 1830 generator, tungsten lamp, and Cu anode, where the source 140 of X-ray is given by a Cu-Kα radiation (λ = 0.15418 nm).

Antibacterial activity of the samples was studied using Escherichia coli (ATCC 25922). A bacterial cell suspension of 10 × 10^5^ CFU/mL was produced by diluting the bacterial culture in distilled water. *E. Coli* was inoculated into a TBX medium (tryptone bile X-gluc) (Liofilchem, Roseto degli Abruzzi TE, Italy). Subsequently, the samples were sterilized and incubated with bacteria for 24 h at 44 °C. Microbial growth was assessed by observing the formation of the colony-forming units. The results were obtained on samples analyzed three times and used to determine the mean standard (SD) deviation of measurements.

## 3. Results and Discussion

**Figure 2:**
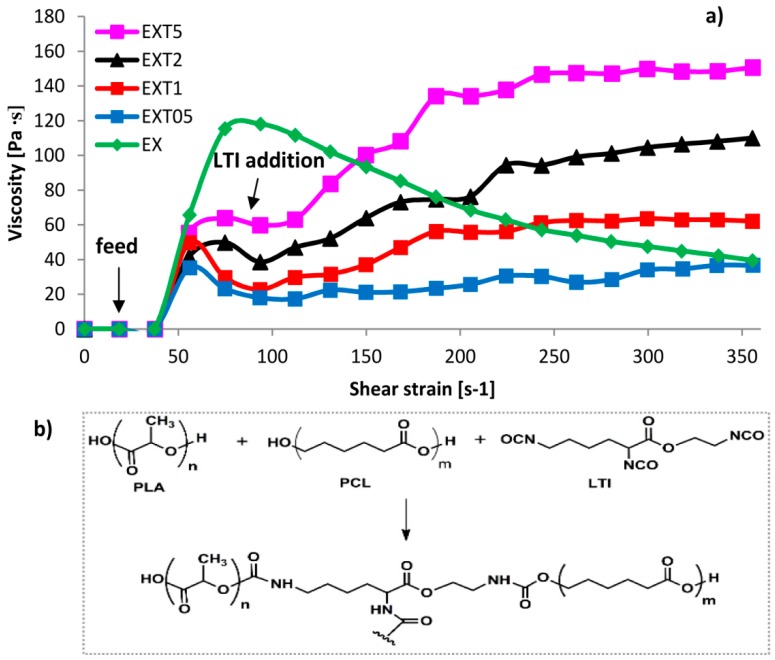
The shear strain of the extruded blends (**a**) with the PLA/PCL/LTI reaction scheme (**b**).

Figure 2a shows the viscosity, η, *vs* the shear strain,γ’, of the extruded blends, without and with different amounts of compatibilizer (LTI). As known, the shear stress depends on the viscosity of polymers, following Newton’s law (τ = η γ’) [30]. After the starting feed period, the viscosity of EX (uncompatibilized blend) is ~120 Pa∙s and then it decreases during the processing of a thermoplastic polymer up to the value of ~40 Pa∙s at higher deformation strains (γ’ = 355 s^−1^) (for the pseudo-plastic behavior). In contrast, compatibilized blends have a different rheological behavior: the starting viscosity is lower than that of the uncompatibilized blend (ranging within 40–60 Pa∙s) for the addition of the liquid LTI which initially plasticizes the melt; than the LTI starts to react with the PCL and PLA, improving the melt viscosity. The viscosity increases with the LTI amount, up to ~150 Pa∙s in the EXT5 sample (at γ’ = 350 s^−1^), resulting in a value 275% higher than that of EX and EXT05 (which is ~40 Pa∙s).

Rheological results clearly suggest a change in the structural organization of the PLA/PCL/LTI blend after LTI addition because the melt viscosities progressively grow with the increasing compatibilizing amount, as indicated by the chemical scheme in Figure 2b. The reactive mixing inside the extruder let to the reaction between PCL and PLA with the three functional groups of LTI producing branched co-polyester urethanes. In accordance with Nocita et al. [31], branched macromolecular chains are formed with an LTI amount ≥1.0phr, while LTI amounts lower than 1 phr are not enough to obtain appreciable viscosity improvements. The viscosity value of the EXT05 blend (which contains 0.5 phr of LTI) is indeed very close to that of the uncompatibilized blend (η= 150Pa∙s, at γ’ = 350 s^−1^). Besides, high amounts of LTI (such as 2.0 phr and 5.0 phr) even produce a 3D cross-linked structure because LTI tends to saturate all the possible reactive sites; at a shear strain of 355 s^−1^, the highest viscosity improvement occurs in the EXT5 blend, where the viscosity increases by 278% with respect to the EX and EXT05 blend.

**Figure 3:**
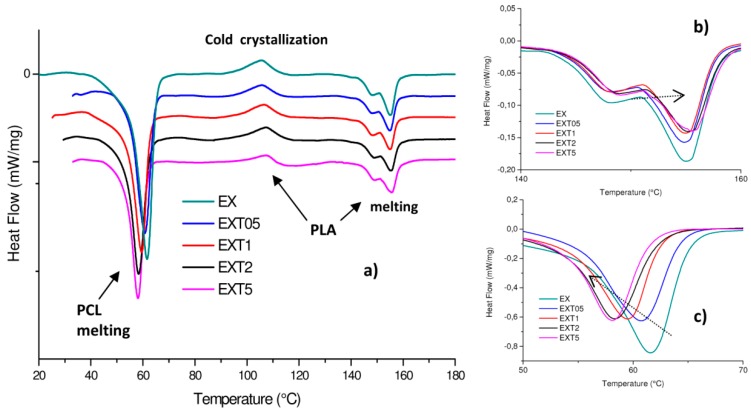
The analysis of blended materials (**a**); magnification of the PLA (**b**) and PCL (**c**) melting range.

Generally, the morphology and phase structure of a blend after thermal treatment can depend on the blending method and crystallization temperature, besides the molecular features of the constituent polymers. Urquijo et al. [32] highlighted the crystallization dynamics of PLA and PCL as the key factor in determining the blend’s resulting mechanical behavior despite the thermo-mechanical processing method used to manufacture the mixtures. Because of the importance of this issue, we investigated the crystallization dynamics of the PCL and PLA of our extruded blends by means of DSC calorimetric analysis, represented in Figure 3a. PLA shows two phase transitions which are associated with melting (composed of two peaks due to the initial D/L mixture poly-lactide crystalline order) and to the cold crystallization, provoked by heating during the calorimetric test. PLA is indeed known for its ability to crystallize after applying heat [33]. DSC parameters (melting temperature and enthalpy, *T_m_* and Δ*H_m_*, cold crystallization temperature and enthalpy, *T_cc_* and Δ*H_cc_*, crystalline degree *χ*_PLA_, *χ*_PCL_, *χ_cc_*_PLA_) are given in detail in Table 2. Our DSC findings for the PLA/PCL (50/50 *w*/*w*) blend are comparable with data obtained by Tsuji et al. [28]. These authors, indeed, for the same blend processed with a shear rate of 3.2 × 10^2^ s^−1^ (and before enzymatic degradation), found a PCL *T_m_*~59 °C, a PLA *T_m_*~168 °C and *T_cc_*~102 °C. The consequent crystalline degree is about 50% for PCL and about 2.2% for PLA.

Calorimetric data highlight the LTI presence changes in the crystalline order of both PCL and PLA. In particular, the *T_m_* value of PCL in all the blends decreases with increasing LTI content from ~61 °C to ~58 °C (Figure 3c), and the corresponding *ΔH_m_* decreases from ~38 to ~25 J/g. The crystalline degree decreases from 53.55% (in EX blend) to 35.24% (in EXT5 blend).

The T_m_ value of PLA in all the blends slightly increases with increasing the LTI content (from 147.9 °C and 155.0 °C, to 149 °C and 155.6 °C, see Figure 3b). The corresponding Δ*H_m_* decreases from 7.45 to 5.98 J/g, see). The crystalline degree of PCL and PLA changes from 53.55% to 35.24% and from 14.99% to 11.00%, in the EX blend and in EXT5 blend, respectively. Thus, PCL has a higher crystalline order than PLA in all the blends and the crystalline order decreases with the increasing LTI amount.

These results confirm that LTI addition causes a micro-structural re-organization in both PLA and PCL according to the reaction scheme of Figure 2 discussed earlier. The decreases in crystalline order are due to the chemical bonds that LTI creates between the PCL and PLA macromolecular chains, forming the co-polyester-urethanes organized in a grafted and/or cross-linked structure, according to the rheological results discussed earlier. The cold crystallization process occurs in all the blends, but this process is more pronounced in the blends with a low LTI amount (such as 0.5 and 1.0 phr, in which the *χ_cc_* is 3.13% and 3.12%) than in those with higher LTI amounts (2.0 and 5.0 phr) in which the *χ_cc_* progressively decreases to 2.9% and 2.13%, again suggesting a different macromolecular organization.

**Figure 4:**
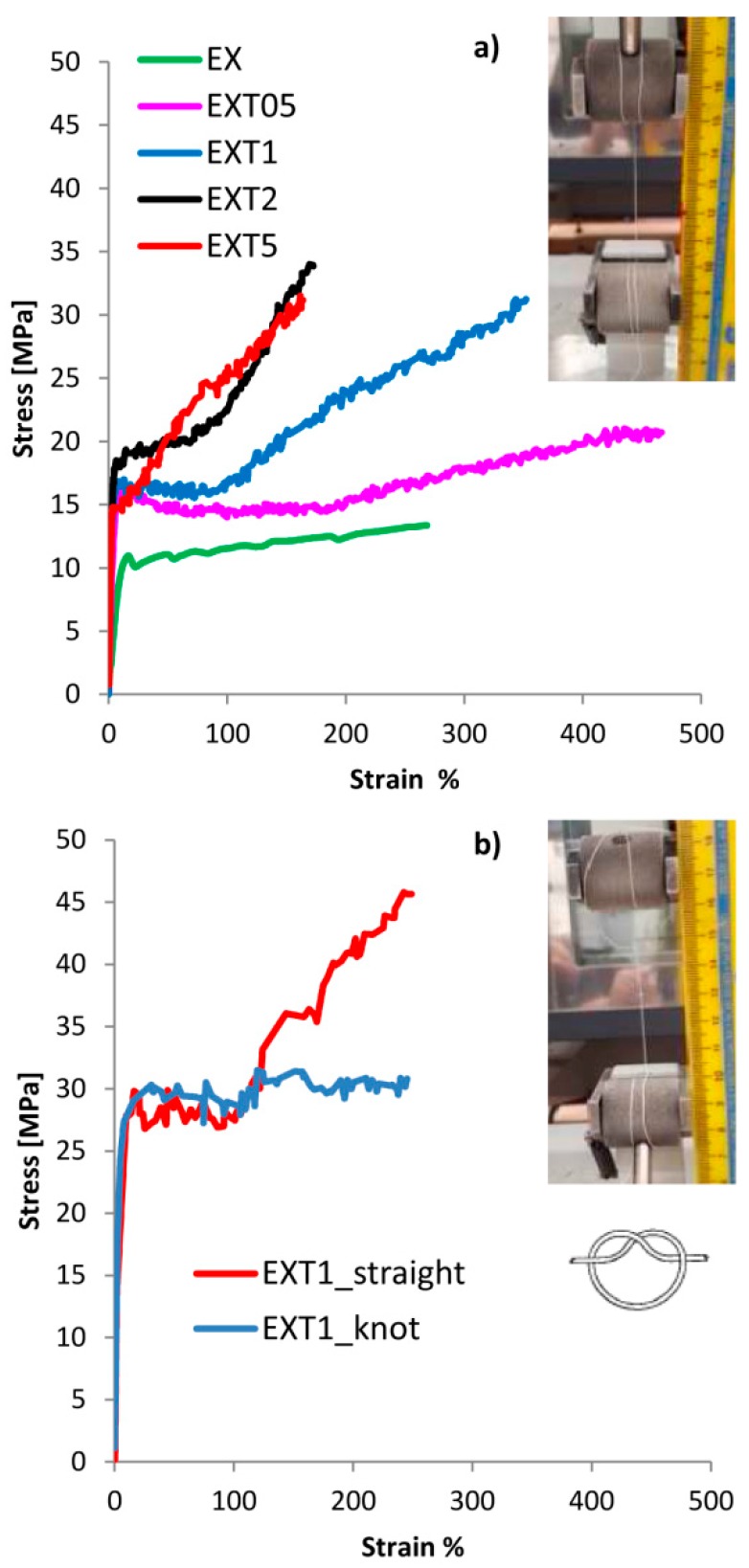
The representative curves of all the blends as ASTM D2256-2002 (**a**), and of the EXT1 thread in straight and in knot situations as the USP standard (**b**).

In order to correlate the different crystalline organizations to the macroscopic behavior of the blended materials, mechanical tensile tests have been performed. Results are plotted in Figure 4a where an image of a thread before the test is also shown.

In Table 3 all the mechanical parameters of the threads are summarized. PCL is much more ductile than pure PLA since their Young moduli are ≈95 MPa and ≈400 MPa, respectively, and their breaking strains are 1470% and 18%, respectively. The yielding strengths of PCL and PLA were about 6 MPa and 23 MPa, and the breaking strengths were 24 MPa and 23 MPa, respectively. Graphs are not shown here for brevity.

As expected, 50/50 (*w*/*w*) blended materials have an intermediate behavior between pure PCL and PLA. All the parameter changes, generally growing and reaching a maximum value of yielding strain (9%), breaking strain (466%) and work at break (7.8 J) in the EXT05 blend, and of yielding strength (18 MPa), breaking strength (34 MPa) and Young Modulus (675 MPa) in the EXT2 blend. Mechanical results suggest that the optimal LTI load is between 0.5 phr and 2.0 phr of LTI, reasonably around 1.0 phr to have a ductile behavior. In blends with an amount of LTI lower than 1.0 phr (around 0.5 phr), the branching reactions are still not enough, while in blends with LTI amounts higher than 1.0 phr (around 2.0 phr) there is an excess of branched and/or cross-linked chains inside the blend.

The EXT2 and EXT5 threads exhibit a low ductility and deformability purely because of too much branched and/or cross-linked structure, according to the calorimetric and rheological results.

Thus, the EXT1 sample was chosen as the best thread in order to check its possible mechanical employment as a suture; with this purpose, the tensile test was repeated in straight (unknotted) and in o-knot (knotted) (Figure 4b). The tensile rate was improved from 10 mm/min, as in the ASTM D2256-2002, to 120 mm/min, as in the UPS standard (see Section 2.2).

As expected, the threads exhibited higher yielding and strength values and lower deformability according to the viscoelastic nature of the polymers. The results indicate that the breaking strength in straight is about 45 MPa, much lower than the commercial absorbable and nonabsorbable sutures (ranging within 410 MPa in Chromic Gut and 1380.1 MPa in rapid polyglactin, both absorbable) [20]. Instead, it is comparable with the data from the literature of similar biopolyesters: Vieira et al. found a value of about 18 MPa in a PLA-PCL (90/10) blend [21] and He et al. [16] a value of about 50 MPa in an unknotted PLLA thread for suture applications.

The breaking strength of the knotted thread was of about 30 MPa, lower than commercial absorbable and nonabsorbable sutures (ranging from 234.9 MPa in Chromic gut to 511.6 MPa in Rapid polyglactin, both absorbable) [20]. Our value is instead comparable with the data from the literature of similar biopolyester since He et al. [16] found a value of 50 MPa in a PLLA knotted thread for suture application.

**Figure 5:**
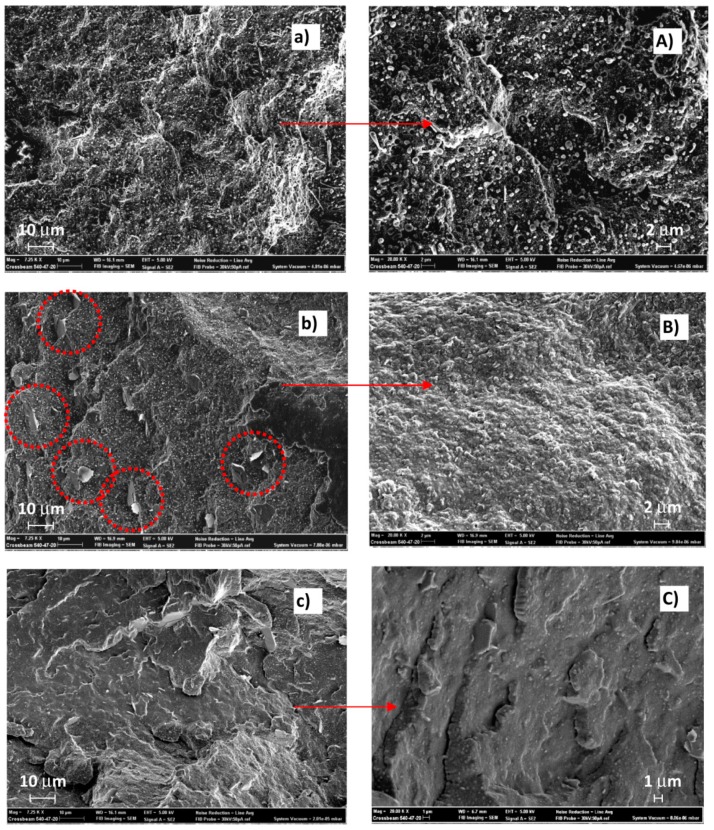
The images at 7250× (left) and at 20k× (right) of threads of EX (**a**,**A**), EXT1 (**b**,**B**), EXT5 (**c**,**C**).

Figure 5 shows SEM micrographs of the thread’s surface broken in nitrogen of the EX blend (uncompatibilized, Figure 5a,A) and of EXT1 (compatibilized with a low LTI amount, Figure 5b,B) and of EXT5 (blend with a high LTI amount, Figure 5c,C), at low (7250×) and high magnification (20k×). The uncompatibilized blend shows a filamentous phase in which small particles, spherical in shape, are dispersed (Figure 5a). The filamentous geometry is due to the preferential longitudinal force direction of the extruded melt. The surface is irregular, suggesting a ductile character of the mixed polymers. Spherical particles of nanometer wideness are detached from the sample’s surface in which several small holes are consequently formed (Figure 5A).

The surface after the compatibilization with 1.0 phr of LTI appears different from the uncompatibilized one since the spherical particles change shape and a filamentous shape appears to be broken into small pieces (see the circles in Figure 5b). The magnification of Figure 5B indicates a uniform polymeric surface, suggesting a good compatibilizing effect of LTI and, hence, a dispersion of the spherical particles in the filamentous matrix. SEM micrographs of the EXT5 sample show a different thread morphology without the micro-irregularity shown in previous images, but with larger agglomerates which appear upon the surface like those evidenced in the circles of Figure 5C.

Furthermore, a smooth surface indicates a brittle behavior of the material. This image morphology is in agreement with the rheological, DSC and mechanical results discussed. Thus, the 1.0 phr of LTI is enough to compatibilize and disperse the two phases retaining a ductile behavior. In contrast, a 5.0 phr of LTI is too high an amount, which brings too many chemical bonds inside the polymeric blend, excessively enhancing the stiffness of the thread.

In order to check the biological features of our threads, bioactivity/biocompatibility tests have been performed on the uncompatibilized and compatibilized samples.

The bioactivity of the samples was evaluated by immersing them in the SBF solution. Kokubo and his colleagues [29,34,35] proposed that one of the important requirements for a good biomaterial is the formation of apatite on its surface after implantation in the human body. Hydroxyapatite nucleation is induced by the interaction between the surfaces of materials and the ions present in the SBF solution, in particular with Ca^2+^, and subsequently with phosphate ions. Ca^2+^ ions combine with the negative charge of phosphate ions allowing the formation of amorphous phosphate, which spontaneously transforms into hydroxyapatite [Ca_10_ (PO_4_)_6_ (OH)_2_] [36].

**Figure 6:**
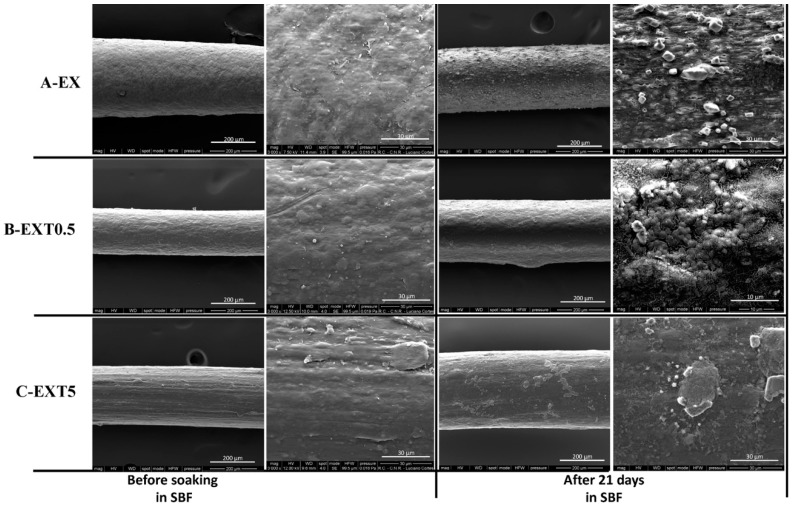
The bioactivity tests of EX (**A**), EXT0.5 (**B**), and EXT5 (**C**) threads.

Figure 6 shows SEM representative images of the threads with different amounts of Triisocyanate, after 21 days in SBF. Comparing all the figures, it is possible to observe a different distribution of the hydroxyapatite globules on their surface. In particular, the low magnification SEM image EXT0.5 after soaking (Figure 6B) shows that the hydroxyapatite layer is not very evident, probably due to the lower thickness of the layer compared to the other threads.

Therefore, the results suggest that the amount of Triisocyanate could influence the formation of hydroxyapatite on the surface of the samples and, thus, their bioactive properties.

**Figure 7:**
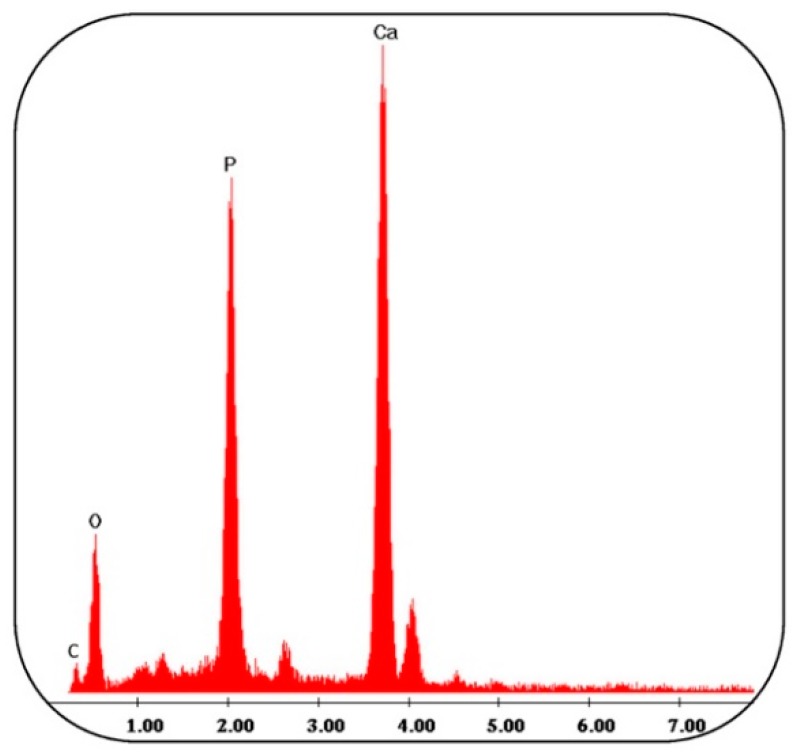
The analysis of the EXT0.5 thread after soaking in a simulated body fluid (SBF) solution for 21 days.

The microanalysis of x-ray (EDX) dispersing energy of the materials was carried out after soaking in SBF for 21 days. The EDX spectra of the different threads were very similar to each other. In Figure 7 a representative EDX spectrum of the EXT0.5 sample is reported, in which it is possible to observe that the globules consisting of Ca and P have an atomic ratio equal to 1.67. The other peaks are residuals of the SBF solution, and, furthermore, it possible to observe the carbon peak originating from PCL and PLA.

**Figure 8:**
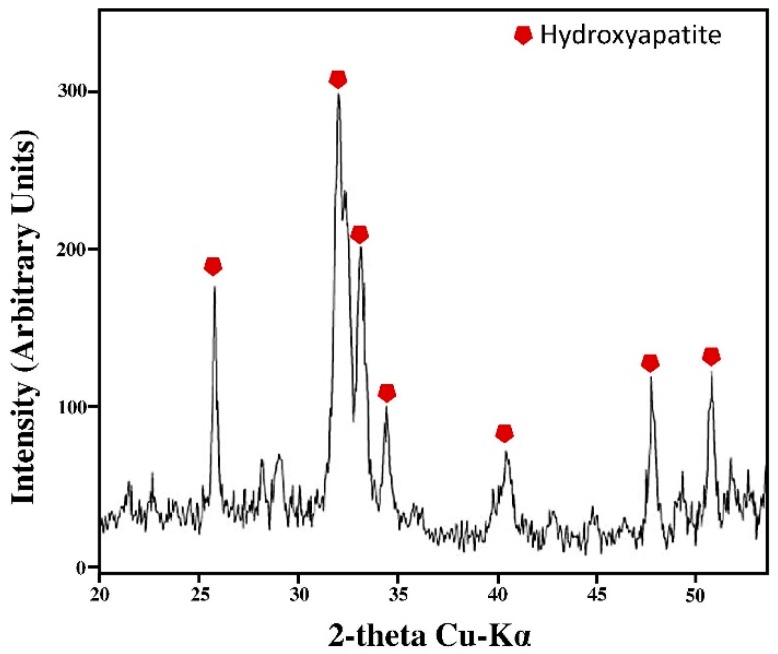
A representative x-ray diffraction (XRD) spectrum of the crystalline hydroxyapatite layer on the EXT0.5 thread surface after soaking in SBF solution for 21 days.

Furthermore, the XRD measurement was used to confirm and identify the presence of hydroxyapatite on the surface of the samples. The intense peaks of crystalline hydroxyapatite are visible after 21 days in SBF (Figure 8). By comparing the XRD pattern of threads before and after immersion in SBF, it can be seen that the peaks in Figure 8 are not present before the soaking in SBF [37], suggesting that they are due to the presence of hydroxyapatite.

The peaks were similar to the phases found in the ICDD database. The main (h k l) indices for hydroxyapatite (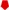) are (002), (211), (300), (202), (310), (002), (222), and (213), regardless of the content of the Triisocyanate. In Figure 8 only the XRD spectrum of the EXT0.5 thread is reported since no significant differences in the intensity of the XRD signals are observed, despite the different amounts of Triisocyanate and the different hydroxyapatite distribution on the material’s surface. The intensity of the XRD signals is due to the hydroxyapatite layer, confirming that the materials are bioactive [38].

The EX, EXT05 and EXT5 samples with different amounts of Triisocyanate were incubated with *E.coli* bacterial strains. Because suture materials are the most frequently used foreign materials in surgeries and since most wound infections occur along suture lines, the use of materials that do not induce bacterial growth is necessary [39].

**Figure 9:**
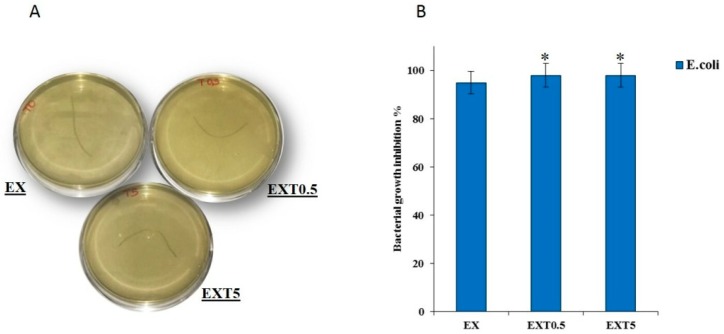
The images of *E. coli* with the EX, EXT0.5 and EXT5 threads (**A**). The bacteria growth inhibition of all samples. Values are the mean standard deviation (SD) of measurements carried out on the samples analyzed three times. The means and SDs are shown. * *p* < 0.05 vs. the bacteria control treated without threads (**B**).

Figure 9A reports representative images of the samples after 24 h of incubation. The antibacterial results show that the materials investigated do not induce the growth of *E. coli*. No significant difference in growth inhibition was found among the different samples. In fact, it is not possible to see the formation of colony-forming units (Figure 9B). Therefore, these results suggest that the presence of these materials in the human body could cause an environment not suitable for bacterial growth. This is probably due to no products that affect the growth and proliferation of bacteria being released from different threads. It was demonstrated that these threads could be used as drug delivery systems. In the future different drugs could be added to reduce the risk of infections.

## 4. Conclusions

In this paper, we studied a blend based on bio-polyesters Poly (Lactic Acid), PLA and Poly (ε-CaproLactone), PCL, with the aim to find a specific application of this material in regenerative medicine. Due to the complementary features of the bio-polyesters, they must be blended and compatibilized with Ethyl Ester l-Lysine Triisocyanate (LTI) at different loads (0.5–1.0–2.0–5.0 phr).

We mixed them in a twin extruder and produced threads by a melt spinning process; the average diameter was about 0.3 mm. In order to check if these threads can be employed as sutures, mechanical tensile (with the calorimetric and morphological investigations), and bioactivity and antibacterial tests are performed.

Results highlighted that the 1.0 phr of LTI seems to be the best compatibilizing amount since lower amounts are enough to appreciably change the blend features, while higher loads produce threads that are too rigid with decreased ductility. This last aspect can be optimized by tailoring the ratio of the two bio-polyesters (i.e., considering the ratio PLA/PCL (20/80) that produces a cross-linked network with a “rubber-like” behavior with a typical high ductility due to a long spacing between the cross-links, already investigated [31]). Thus, the PLA/PCL/LTI threads could be employed as sutures since they were shown to be ductile materials, with appreciable stiffness and deformability even when a knot is formed, and to be bioactive, able to inhibit the bacterial growth. Furthermore, in order to improve the thread processing, extrusion in an inert environment can be performed to avoid possible thermomechanical degradation of PLA. Work is in progress to evaluate another important aspect of their use as absorbable threads, namely, the appropriate time of bio-degradability of the sutures.

## Figures and Tables

**Table 1:** The simulated body fluid (SBF) composition.

Ion	SBF (mol m^3^)	Human Blood Plasma (mol m^3^)
Na^+^	142.0	142.0
K^+^	5.0	5.0
Mg^2+^	1.5	1.5
Ca^2+^	2.5	2.5
Cl^−^	147.8	103.0
HCO_3_^−^	4.2	27.0
HPO_4_^2−^	1.0	1.0
SO_4_^2−^	0.5	0.5

**Table 2:** The DSC data of all the extruded threads.

Thread Sample	Melting Temperatures (°C)				Enthalpy (J)			Crystalline Degree (%)		
	*T_m_* PCL	*T_cc_* PLA	*T_m_* PLA		Δ*H_m_* PCL	Δ*H_cc_* PLA	Δ*H_m_* PLA	*χ* PCL	*χ_cc_* PLA	*χ* PLA
**EX**	61.59	105.45	147.99	155.04	38.02	2.67	7.45	53.55	14.99	3.95
**EXT0.5**	60.77	105.78	147.73	154.89	28.06	2.12	6.32	39.52	12.50	3.13
**EXT1**	59.48	106.62	147.73	154.93	25.40	2.10	5.56	35.77	11.48	3.12
**EXT2**	58.36	107.30	148.55	155.25	25.29	2.00	5.50	35.62	11.11	2.96
**EXT5**	58.09	107.46	149.00	155.60	25.02	1.44	5.98	35.24	11.00	2.13

**Table 3:** The tensile static mechanical test results of all the extruded threads.

Threads Sample	Rate (mm/min)	Yielding Strength (MPa)	Yielding Strain (%)	Breaking Strength (MPa)	Breaking Strain (%)	Young Modulus (MPa)	Work at Break (J)
EX	10	11.0 ± 0.9	16.7 ± 1.2	13.3 ± 0.4	268.4 ± 20.6	151.1 ± 7.3	3.2 ± 0,2
EXT0.5	10	15.9 ± 1.4	9.0 ± 0.6	20.7 ± 1.9	466.4 ± 39.1	407.4 ± 20.7	7.8 ± 0.4
EXT1	10	16.8 ± 0.6	8.4 ± 0.3	31.2 ± 3.1	352.3 ± 25.4	609.2 ± 55.2	7.7 ± 0.2
EXT2	10	18.5 ± 1.7	6.4 ± 0.5	33.9 ± 3.3	172.8 ± 8.6	674.8 ± 63.4	4.1 ± 0.1
EXT5	10	14.8 ± 1.3	3.8 ± 0.2	31.2 ± 2.9	164.0 ± 9.9	461.0 ± 35.1	3.8 ± 0.1
EXT1	120	29.8 ± 2.8	19.4 ± 1.7	45.7± 3.2	244.9 ± 20.4	721.9 ± 65.3	5.0 ± 0.3
EXT1	120-knot	-	-	30.8± 2.1	245.3 ± 14.5	708.4 ± 50.8	4.4 ± 0.2
